# Supplementary material for: In-situ formatting donor-acceptor polymer with giant dipole moment and ultrafast exciton separation
Source: Nat Commun. 2024 Feb 13;15:1313. doi: 10.1038/s41467-024-45604-5 (PMC10864376; doi:10.1038/s41467-024-45604-5)
Supplement: Supplementary file 1 — Supplementary Information [file 41467_2024_45604_MOESM1_ESM.pdf]

Supplementary Information

In-situ formatting donor-acceptor polymer with giant dipole moment and ultrafast exciton separation

Chang Cheng,[1] Jiaguo Yu,[1,2,*] Difa Xu,[3] Lei Wang,[4] Guijie Liang,[4] Liuyang Zhang[2,*] & Mietek Jaroniec[5,*]

Supplementary Figures

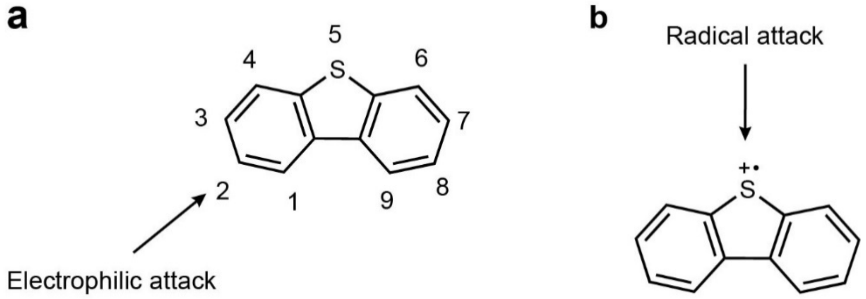

Supplementary Figure 1. Active centers of dibenzothiophene units and their cations. a, Electrophilic attack on dibenzothiophene (DBT) always occurs at 2 (8)-positions. Hence, in this *Friedel-Crafts* polymerization, the chain propagation centers are located at 2 (8)-positions[1]. **b,** S atom is the predominant position for the radical attack by dibenzothiophene cations ($DBT^{+\bullet}$)[2].

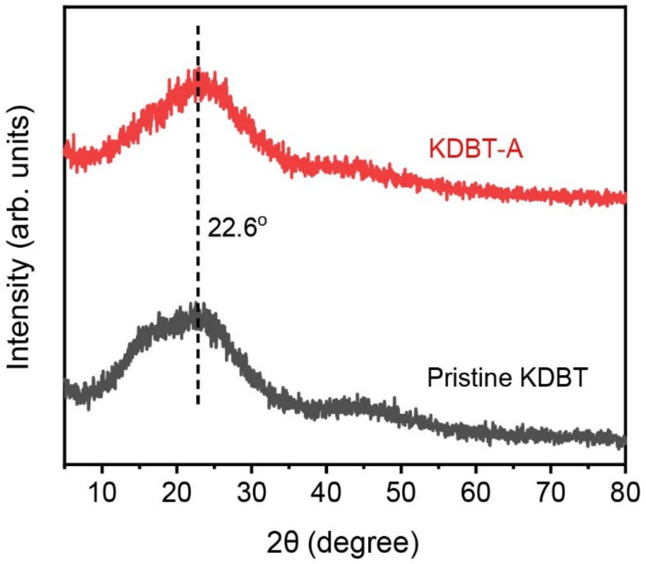

Supplementary Figure 2. Phase structure of the samples. XRD patterns of pristine KDBT and KDBT-A. The hump at 2θ = 20-30° is the typical signal of π-π stacking.

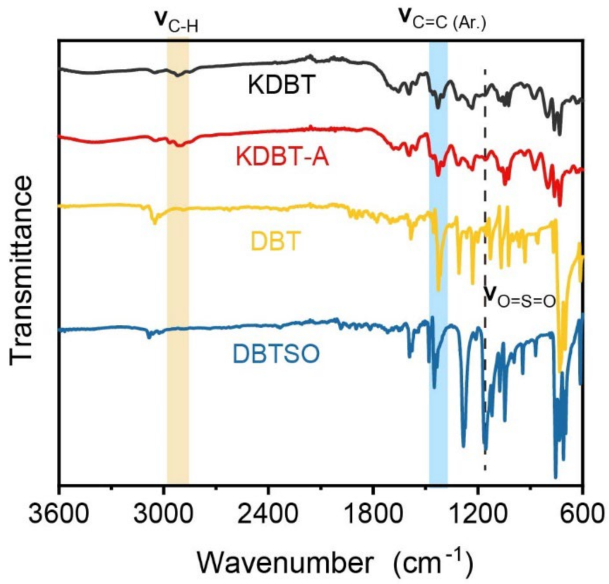

Supplementary Figure 3. FT-IR spectra of pristine KDBT, KDBT-A, DBT, and dibenzothiophene-*S,S*-dioxide (DBTSO). The strong absorption peaks at 1485 and 2920 cm^{-1} belong to the aromatic ring skeleton vibration and C-H stretching vibration, respectively, indicating the successful knitting of DBT units. After long-time irradiation, these peaks are still present, confirming the robust molecular skeleton of the polymer. The peak at 1150 cm^{-1} reflects the characteristic signal of the sulphonyl group (O=S=O), which emerges on the spectra of KDBT-A and implies that DBT units are partly oxidized to DBTSO.

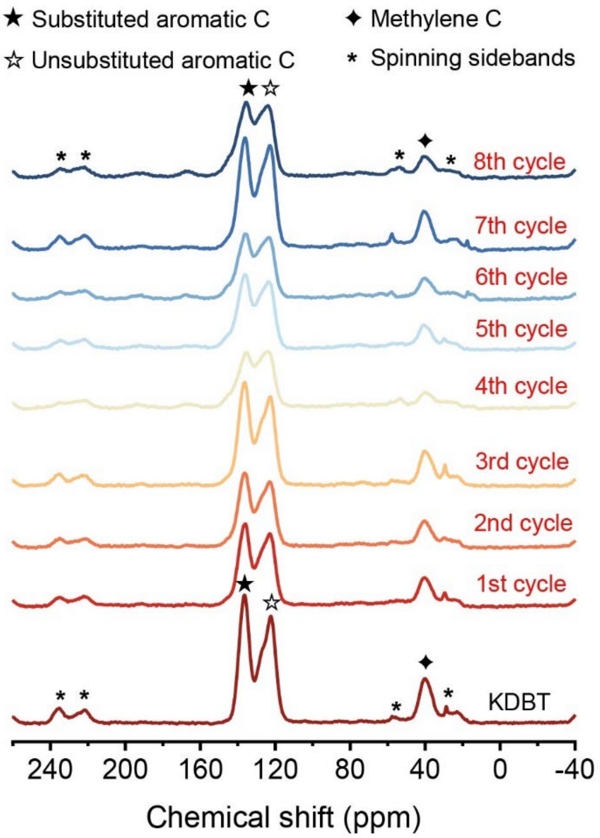

Supplementary Figure 4. Stability of the polymer backbone. ^{13}C CP/MAS NMR spectra of the polymer during the cycle tests. Solid pentagrams denote substituted aromatic carbon atoms, hollow pentagrams designate unsubstituted aromatic carbon atoms, diamonds represent methylene carbon atoms, asterisks denote spinning sidebands.

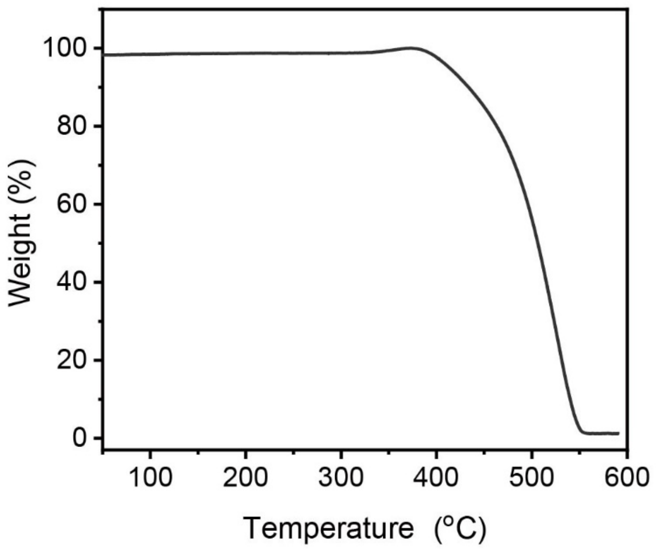

Supplementary Figure 5. Thermal stability of KDBT sample. Thermogravimetric analysis of KDBT at a heating rate of 10 °C/min^{-1} under air atmosphere. The as-synthesized polymer reveals a high decomposition temperature and totally pyrolyzes after 550 °C. Interestingly, the weight of KDBT slightly increases before decomposition, implying that oxygen may enter the molecular backbones.

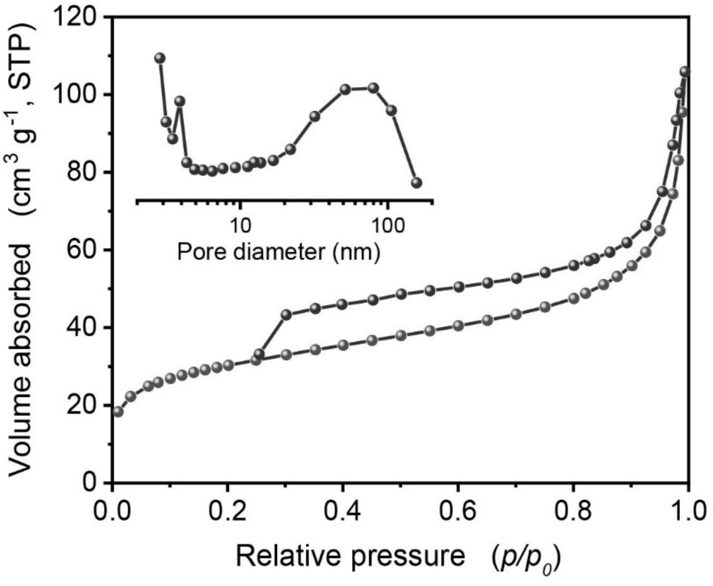

Supplementary Figure 6. Pore structure of KDBT. Nitrogen adsorption-desorption isotherm of KDBT at 77.3 K. The curve exhibits type IV isotherm featuring a typical H4 hysteresis loop at p/p_0 = 0.25–0.95 according to the International Union of Pure and Applied Chemistry (IUPAC) and Brunauer-Deming-Demin-Teller (BDDT) classification, implying the existence of micro- and mesopores in the samples. The inserted pore size distribution verifies the abundant micro-, meso-, and macropores in KDBT. Its surface area and single-point pore volume are 107 m^2 g^{-1} and 0.12 cm^3 g^{-1}, respectively.

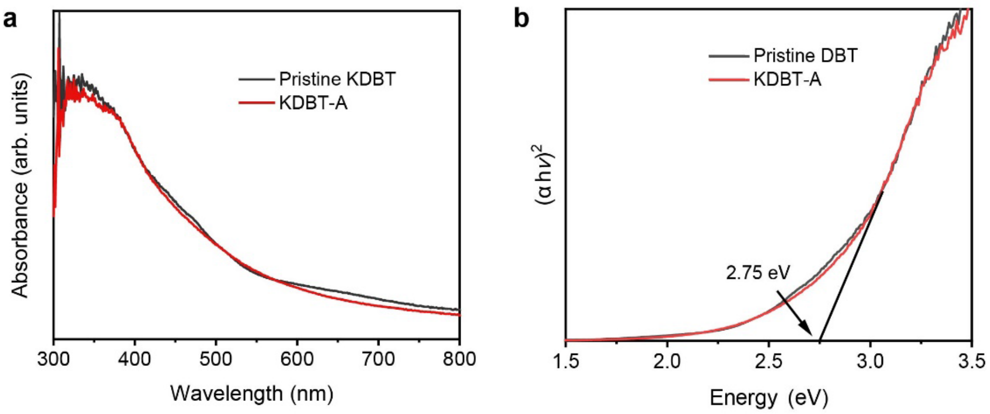

Supplementary Figure 7. Optical absorption properties of the resultant samples. **a,** UV-vis DRS of pristine KDBT and KDBT-A, and **b,** the corresponding Tauc plot. Benefiting from the hyperconjugation effect ($\sigma_{C\text{-}H}\rightarrow\pi^*$), the polymers exhibit broad absorption in the UV-visible light region.

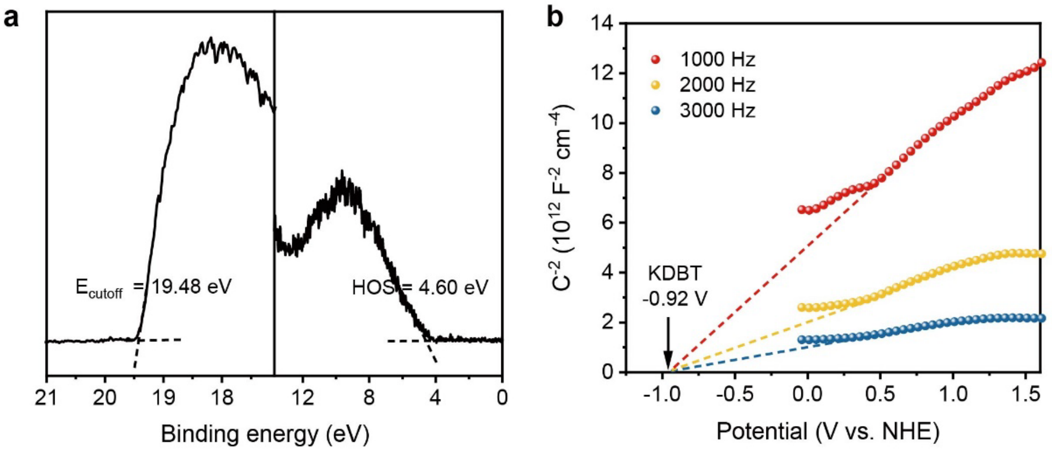

Supplementary Figure 8. Band structures of pristine KDBT. a, Ultraviolet photoelectron spectra of pristine KDBT. According to the values of cutoff and the highest occupied states (HOS), ionization potential (*Ip*, equivalent to valance band vs. Vacuum) could be obtained with the equation of $Ip = 21.22\ \text{eV} - (E_{\text{cutoff}} - HOS)$. **b,** Mott-Schottky plots of KDBT. C refers to the interfacial capacitance. Experimental conditions: the sample drop-coated glassy carbon electrode as the working electrode, Ag/AgCl electrode as the reference electrode, a platinum sheet as the counter electrode, 0.5 M Na_2SO_4 solution as the electrolyte.

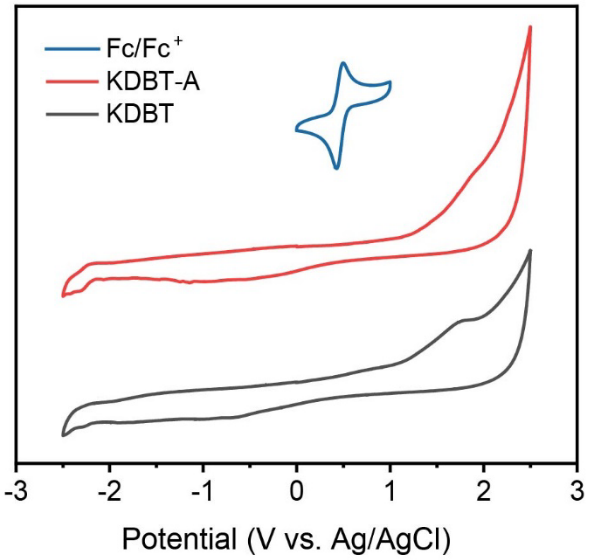

Supplementary Figure 9. Band structures obtained from cyclic voltammetry (CV) tests. The CV curves of ferrocene, KDBT, and KDBT-A. Ferrocene (Fc) served as an internal standard with an assigned absolute energy of −4.8 eV vs. the vacuum level.

The HOMO levels (vs. vacuum) were calculated by the following Supplementary Equation (1):

$$E_{\mathrm{HOMO}} = -\mathrm{e} \times (E_{\mathrm{ox}} + 4.8 - E_{\mathrm{ox}}^{\mathrm{Fc/Fc^+}}) \qquad (1)$$

where E_{ox} and $E_{\mathrm{ox}}^{\mathrm{Fc/Fc^+}}$ are the onset oxidation potential of polymers and ferrocene vs. Ag/AgCl.

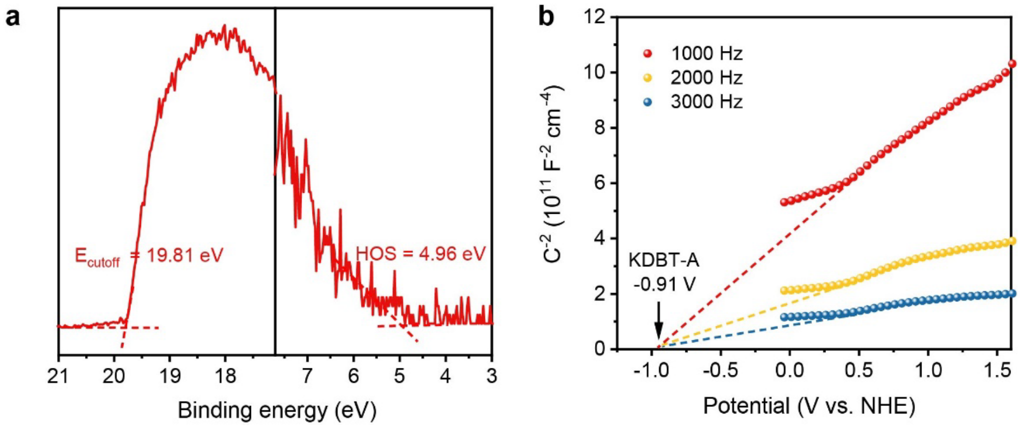

Supplementary Figure 10. Band structures of KDBT-A. a,b, Ultraviolet photoelectron spectra and Mott-Schottky plots of KDBT-A.

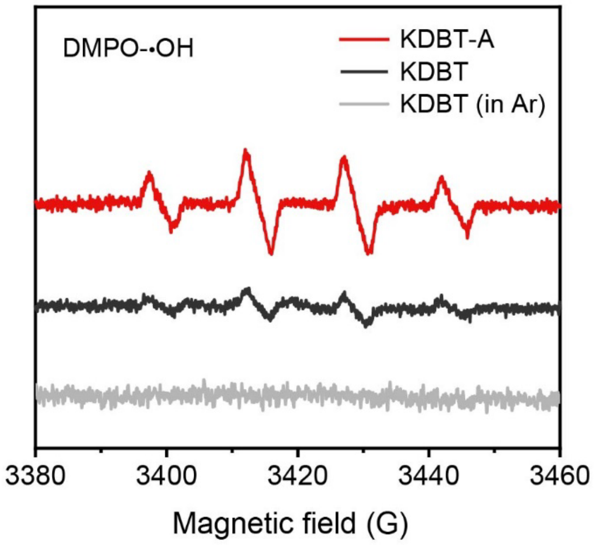

Supplementary Figure 11. Electron paramagnetic resonance (EPR) analysis of the samples. Light-irradiated EPR spectra of DMPO-•OH adducts. Experimental conditions: 2 mg of samples, 10 mL of ultrapure water, LED (λ = 365 nm) irradiation for 5 min.

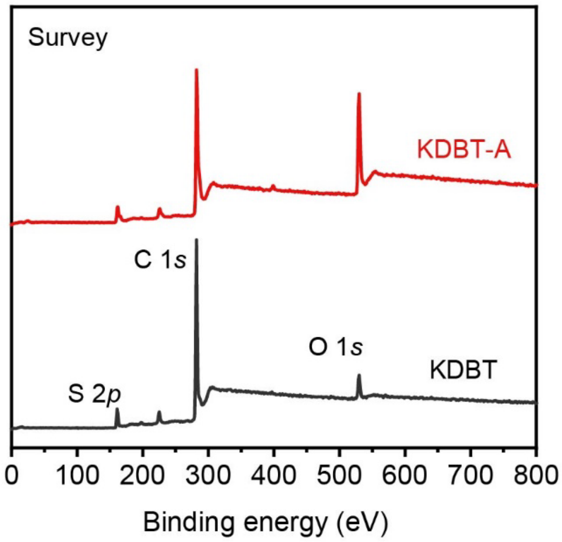

Supplementary Figure 12. XPS survey spectra of the samples. The S/C atomic ratio of KDBT is close to that of KDBT-A, indicating the robust polymer skeleton. The O 1*s* peak is significantly amplified in KDBT-A, suggesting the incorporation of O atoms into polymer chains.

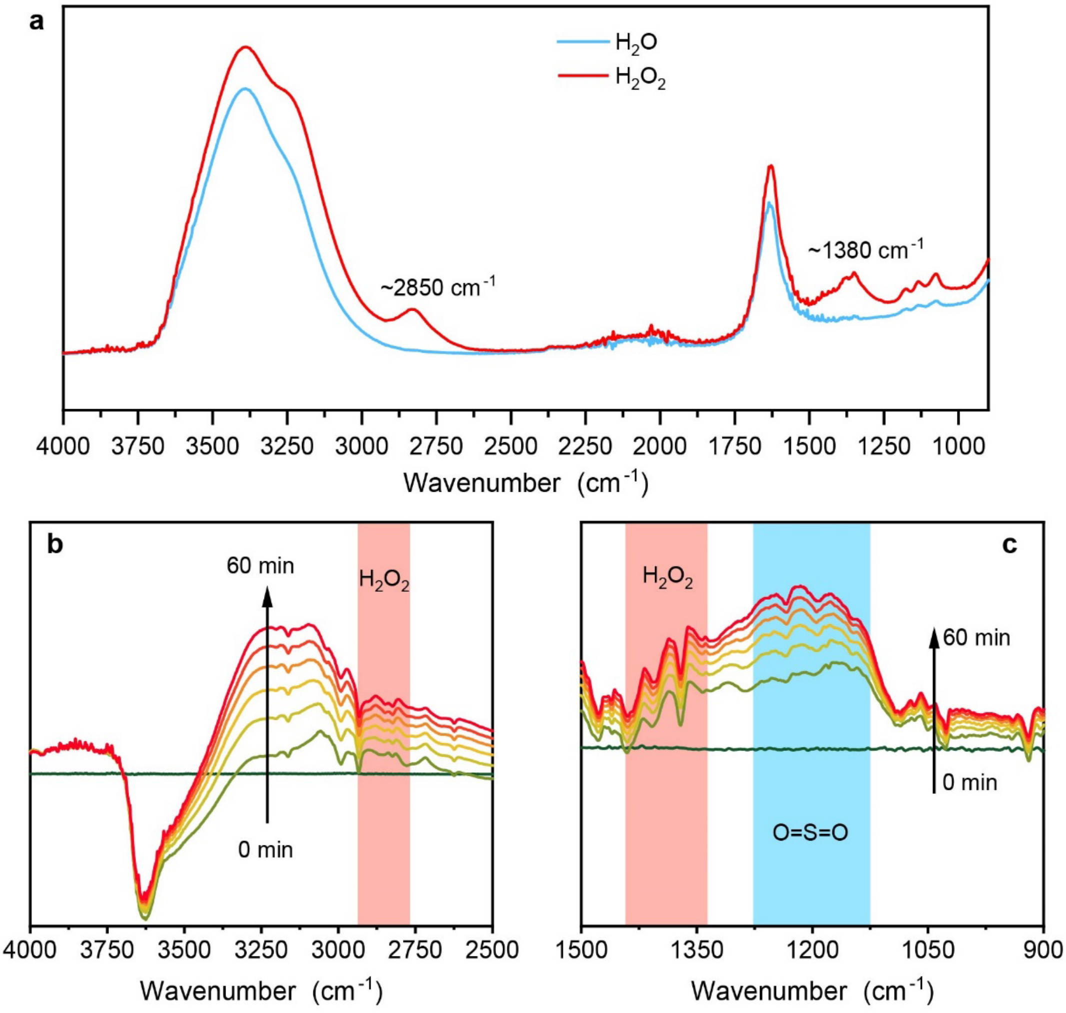

Supplementary Figure 13. FTIR and In-situ DRIFTS spectra. a, FTIR spectra depicting comparisons between H_2O and H_2O_2. **b,** In-situ DRIFTS spectra showcasing signals within the 2500-4000 cm^{-1} range. **c,** In-situ DRIFTS spectra illustrating signals spanning 900-1500 cm^{-1} range.

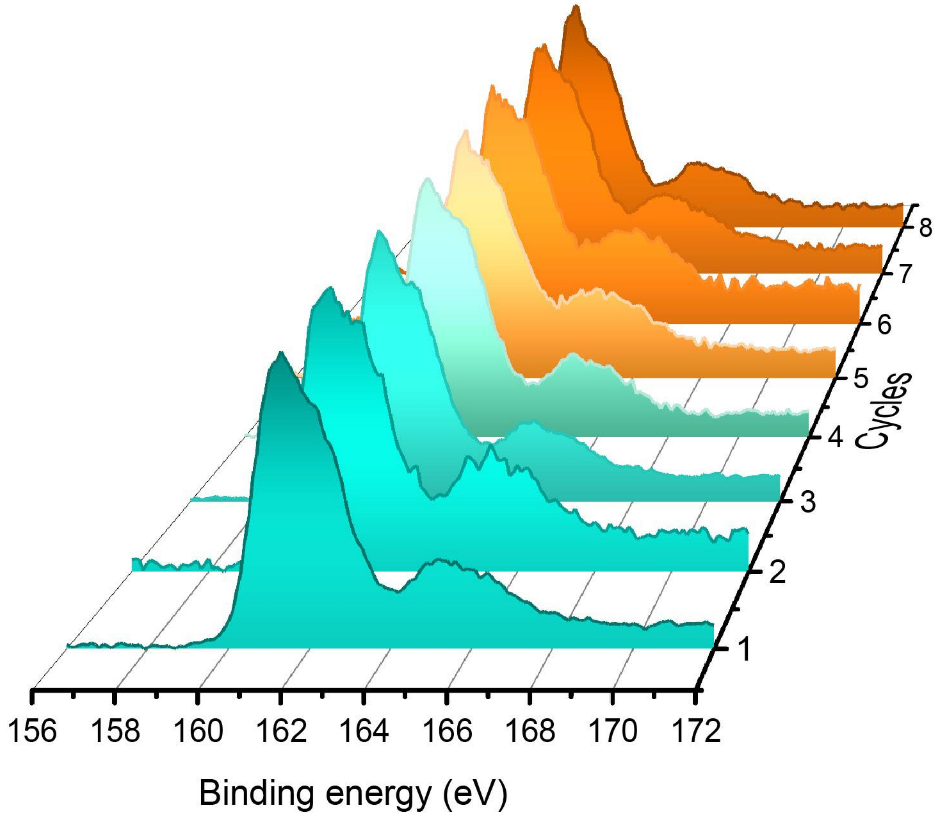

Supplementary Figure 14. Stability of the sulphonyl group. S 2*p* XPS spectra of the polymer during the cycle tests. The percentage of the sulphonyl group S 2*p* signal remained within the range of 25% to 30%.

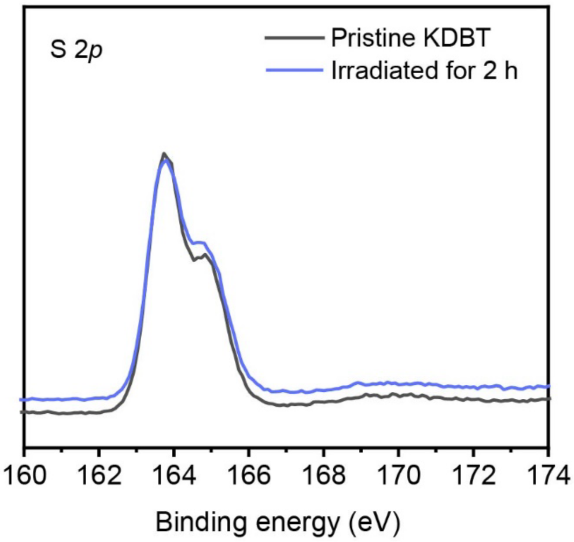

Supplementary Figure 15. High-resolution XPS spectra of S 2*p* in KDBT before and after irradiation. Reaction conditions: 5 mg of photocatalyst, 30 mL of 10 v.% methanol aqueous solution, and an air atmosphere. After 2 hours of irradiation, there is no oxidation of the DBT unit.

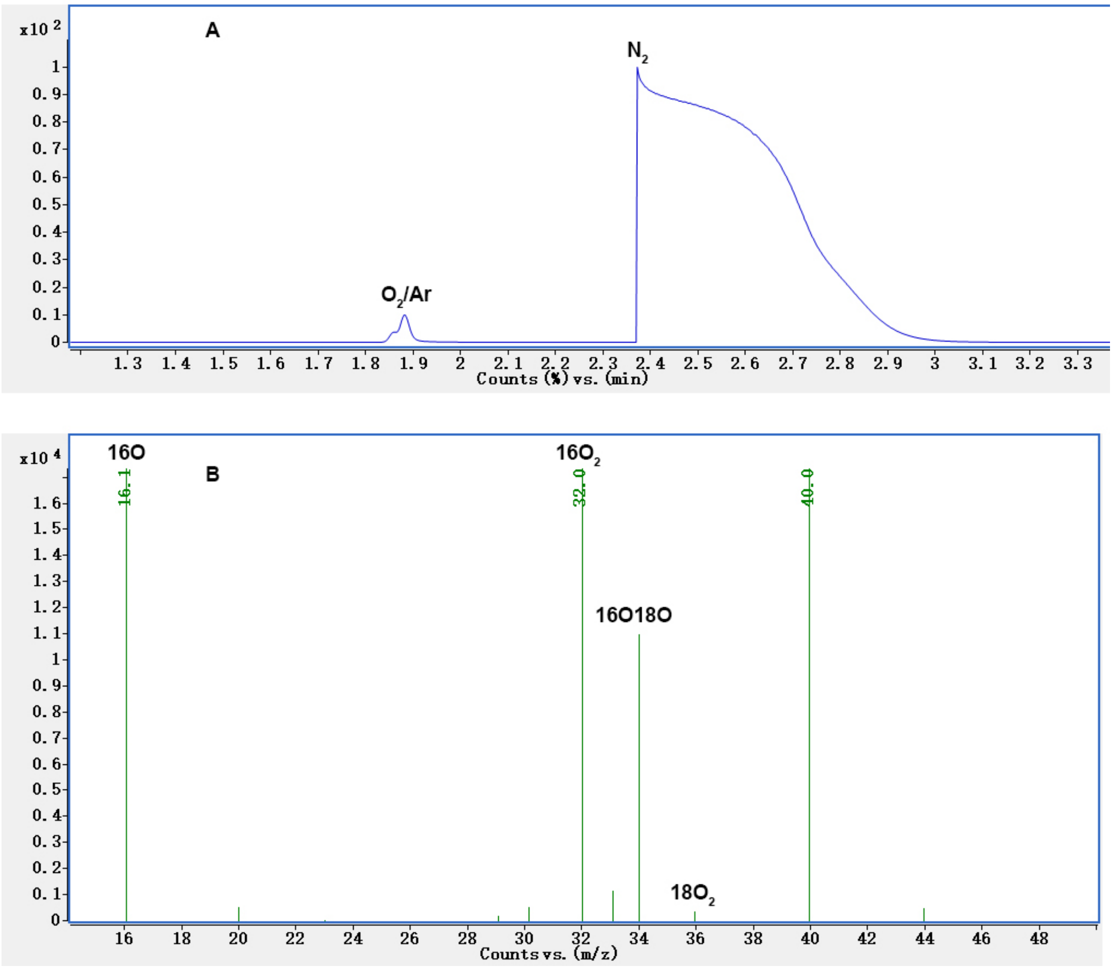

Supplementary Figure 16. The isotopic analysis of oxidation half-reaction on KDBT using $H_2{}^{18}O$. a, Total ion chromatography. **b,** Mass spectra of O_2/Ar signal.

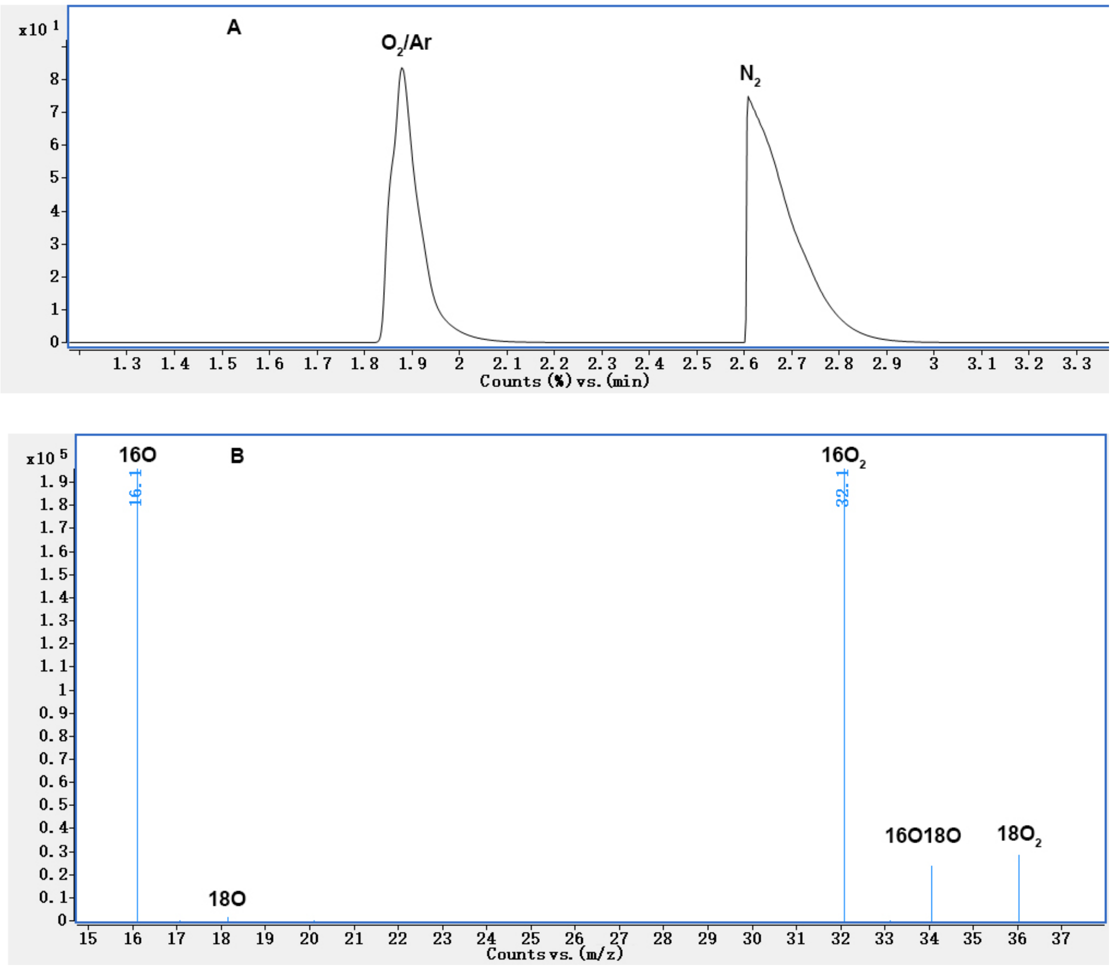

Supplementary Figure 17. The isotopic analysis of oxidation half-reaction on KDBT-A using $H_2{}^{18}O$. a, Total ion chromatography. **b,** Mass spectra of O_2/Ar signal.

Photocatalytic mechanism

Based on the control experiments and isotopic analysis, the H_2O_2 generation mechanism is presented as follows. Whether KDBT is oxidized or not, superoxide radicals ($\bullet O_2^-$) are the primary active species. Hence, H_2O_2 is mainly photosynthesized via the two-electron oxygen reduction reaction ($2e^-$ ORR, Supplementary Equation (2) and Supplementary Equation (3)). While the photogenerated holes undergo a four-electron water oxidation reaction ($4e^-$ WOR, Supplementary Equation (4)) to generate O_2[3].

$$\mathrm{O_2} + \mathrm{e^-} \rightarrow \cdot \mathrm{O_2^-}, \qquad E_{\mathrm{redox}} = -0.33\ V_{\mathrm{NHE}} \tag{2}$$

$$\cdot \mathrm{O_2^-} + \mathrm{e^-} + 2\mathrm{H^+} \rightarrow \mathrm{H_2O_2}, \qquad E_{\mathrm{redox}} = +1.44\ V_{\mathrm{NHE}} \tag{3}$$

$$2H_2O + 4h^+ \rightarrow O_2 + 4H^+, \qquad E_{redox} = +1.23\ V_{NHE} \qquad (4)$$

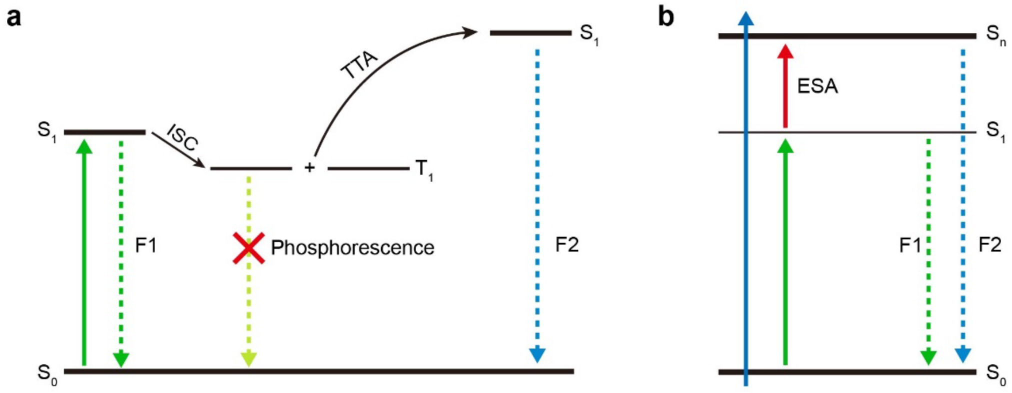

Supplementary Figure 18. Possible photophysical processes. a,**b**, Schematic illustration of triplet-triplet annihilation upconversion (TTA-UC) (**a**) and excitation/relaxation procedures of singlet excited states (**b**). The TTA-UC pathway is not relevant since no triplet excited state (phosphorescence emission) is detected. In the second process, polymer molecules transit to the first singlet excited state ($S_0 \rightarrow S_1$) and then to a higher singlet excited state ($S_1 \rightarrow S_n$).

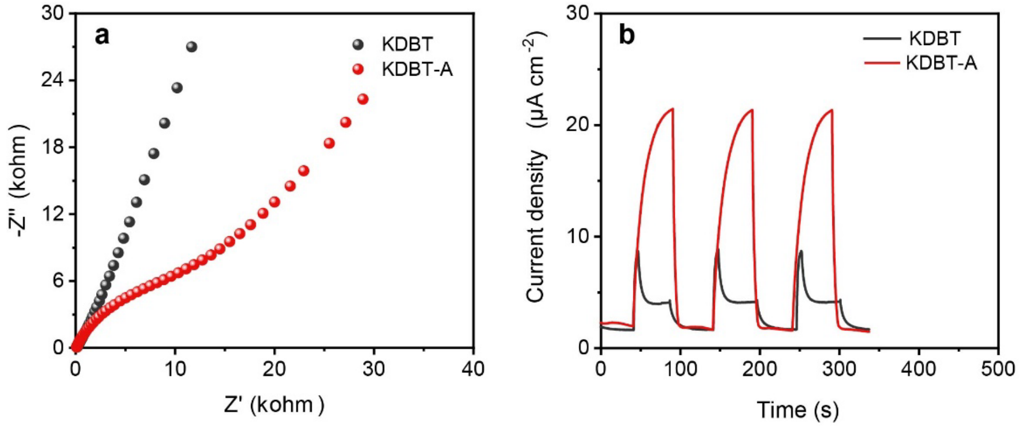

Supplementary Figure 19. Electrochemical measurements. a,**b**, EIS Nyquist plots and photocurrent curves of KDBT and KDBT-A. Experimental conditions: The samples coated on Fourine-doped tin oxide (FTO) glass were used as the working electrode, an Ag/AgCl electrode as the reference electrode, a platinum sheet as the counter electrode, and 0.5 M Na_2SO_4 solution as the electrolyte. KDBT-A exhibits a smaller arc radius of Nyquist plots and higher photocurrent density than KDBT, indicating its more efficient charge mobility and separation.

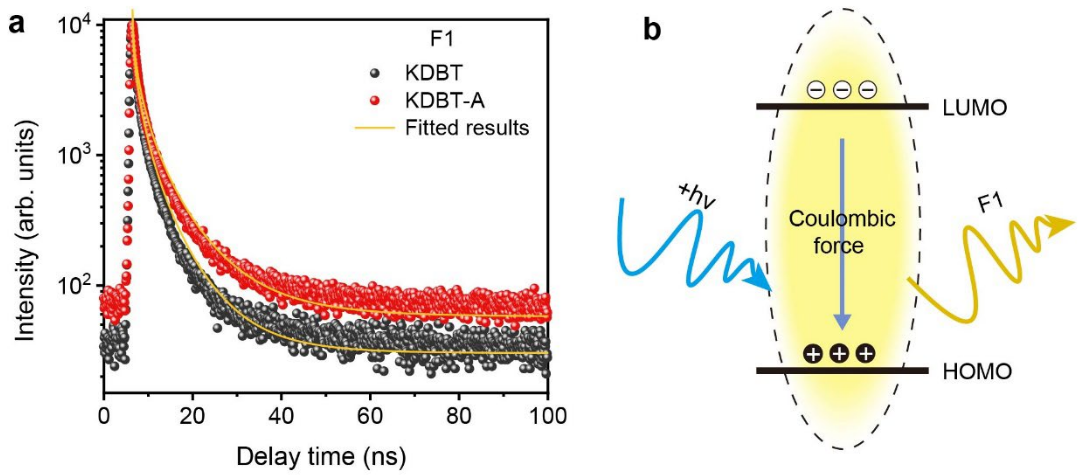

Supplementary Figure 20. Decay dynamics of fluorescence F1 peak. a, Time-resolved fluorescence spectra of KDBT and KDBT-A under an argon atmosphere. The emission wavelength was fixed at 528 nm for KDBT and 566 nm for KDBT-A; hence, the decay kinetics reflects the recombination of photogenerated electron-hole pairs. **b**, Schematic illustration of the process.

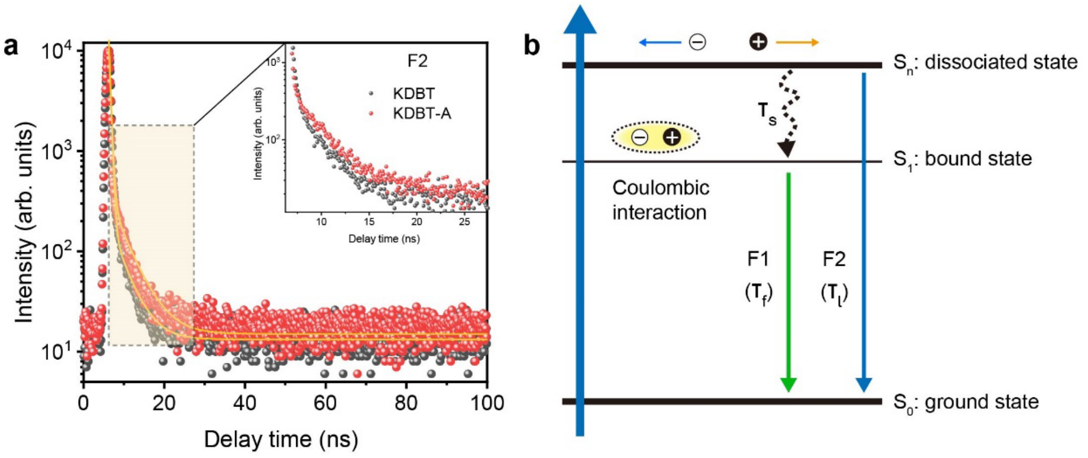

Supplementary Figure 21. Decay dynamics of fluorescence F2 peak. a, Time-resolved fluorescence spectra of KDBT and KDBT-A at $\lambda_{emission}$ = 428 nm. Inset: zoom-in decay of fluorescence F2 peak in the first 30 ns. **b,** Two pathways dominate the attenuation of separated excitons.

The decay curves can be well-fitted by the following multi-exponential Supplementary Equation (5):

$$I_{(t)} = I_{(0)} + \sum_{i=1}^{n} A_i \exp\left(-t/\tau_i\right) \qquad (5)$$

where I_0 represents the baseline correction value, and t is the probe time delay. A_i and τ_i are amplitudes and decay times, respectively. To satisfactorily fit the experimental data, the minimum number of components n is one for F1 and two for F2 peaks.

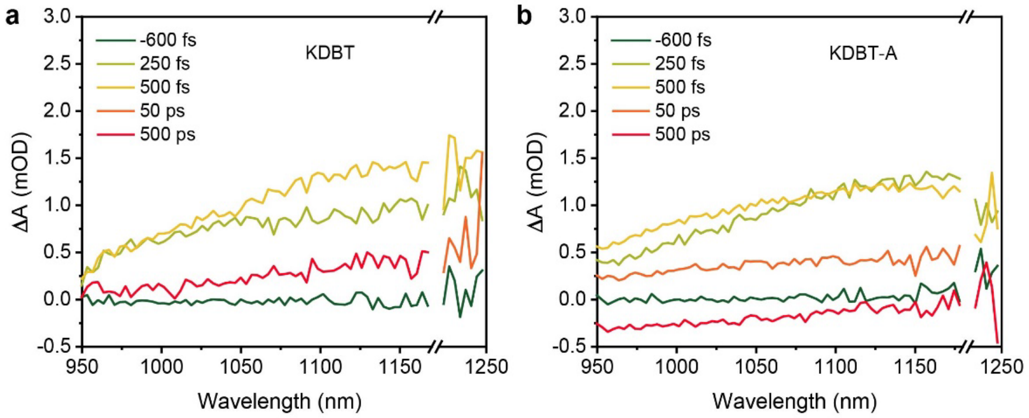

Supplementary Figure 22. Representative transient absorption spectra signals. **a**,**b**,Transient absorption spectra of KDBT and KDBT-A. The change in absorbance (ΔA) is defined as the optical density (OD) difference.

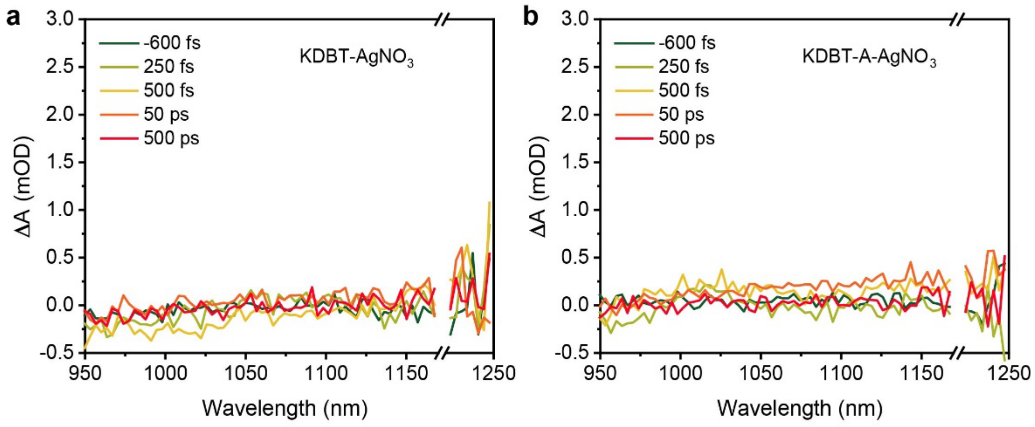

Supplementary Figure 23. Representative transient absorption spectra signals. **a**,**b**,Transient absorption spectra of KDBT and KDBT-A in the presence of $AgNO_3$ as electron scavengers. The mass ratio of photocatalysts to $AgNO_3$ is 1:1.

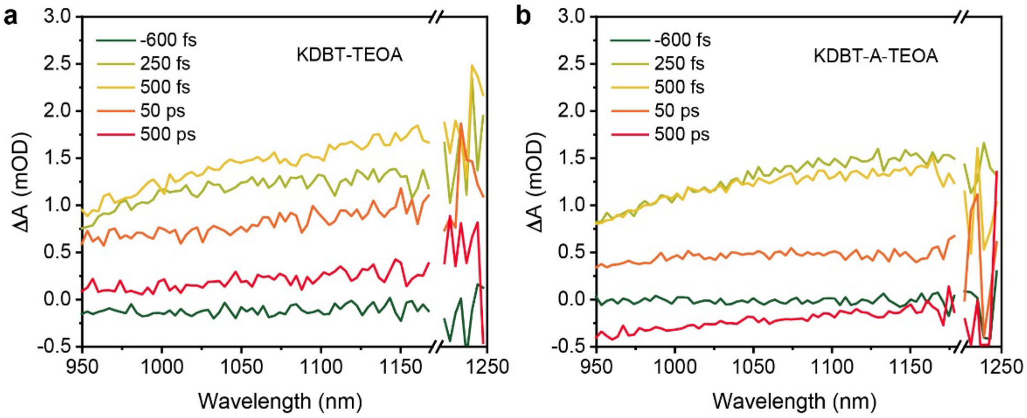

Supplementary Figure 24. Representative transient absorption spectra signals. a,**b**,Transient absorption spectra of KDBT and KDBT-A in the presence of TEOA as hole scavengers. The concentration of TEOA is 10 v.%.

Supplementary Tables

Supplementary Table 1. The band structures of KDBT and KDBT-A obtained from ultraviolet photoelectron spectroscopy (UPS), Mott-Schottky (M-S) measurements, and cyclic voltammetry (CV) tests.

Sample	UPS (*V* vs. NHE)		M-S (*V* vs. NHE)		CV (*V* vs. NHE)	
	HOMO	LUMO	HOMO	LUMO	HOMO	LUMO
KDBT	+1.84	−0.91	+1.83	−0.92	+1.75	−1.00
KDBT-A	+1.87	-0.88	+1.84	−0.91	+1.86	−0.89

Supplementary Table 2. Comparative analysis of photocatalytic H_2O_2 production activity between the present work and other polymer photocatalysts.

Photocatalyst	Condition	Rate (μM h^{-1})[a]	Ref.
CHF-DPDA	O_2	3450	4
TTF-BT-COF	O_2	1380	5
N_0-COF	O_2	785	6
RF523	O_2	138	7
Bpy-TAPT	O_2	686	8
CTF-BDDBN	O_2	58	9
HEP-TAPT-COF	O_2	875	10
MRF-250	O_2	972	11
6DEPI	O_2	97	12
TDB-COF	O_2	724	13
KDBT	Air	870	This work
KDBT-A	Air	1320	This work

[a] The unit of H_2O_2 production rate is standardized to μM h^{-1}.

Supplementary Table 3. Effect of monomer concentration and duration of ultrasonication on the morphology of KDBT polymer.

Conditions		Time[b]		
		2	3	4
Conc.[a]	0.02	particle	particle/tube	sphere/tube
	0.025	sphere/tube	tube	tube
	0.05	sphere	sphere/tube	bulk

[a] Concentration of DBT units (mol/L).

[b] Duration of Ultrasonication (h).

Supplementary Table 4. Input geometry of DBT-carbon-DBT fragments.

Atom	x	y	z
S(1)	0.512	1.726	2.291
C(2)	1.113	1.312	1.016
C(3)	0.438	0.319	0.417
C(4)	-0.611	-0.007	1.181
C(5)	-0.608	0.777	2.268
C(6)	2.215	1.822	0.445
C(7)	2.626	1.32	-0.732
C(8)	1.946	0.325	-1.328
C(9)	0.843	-0.186	-0.757
C(10)	-1.562	-0.932	0.995
C(11)	-2.533	-1.078	1.917
C(12)	-2.524	-0.283	3.004
C(13)	-1.57	0.645	3.192
S(14)	-8.216	0.497	-0.349
C(15)	-7.096	-0.182	0.316
C(16)	-6.068	-0.429	-0.508
C(17)	-6.375	0.033	-1.725
C(18)	-7.601	0.575	-1.681
C(19)	-7.027	-0.549	1.604
C(20)	-5.915	-1.164	2.042
C(21)	-4.879	-1.42	1.221
C(22)	-4.961	-1.044	-0.069
C(23)	-5.664	0.015	-2.861
C(24)	-6.207	0.555	-3.965
C(25)	-7.436	1.098	-3.927
C(26)	-8.144	1.113	-2.784
C(27)	-3.63	-2.104	1.732
H(28)	2.789	2.638	0.917
H(29)	3.53	1.728	-1.214
H(30)	2.297	-0.075	-2.294
H(31)	0.293	-1	-1.254
H(32)	-1.557	-1.567	0.096
H(33)	-3.319	-0.39	3.761
H(34)	-1.598	1.277	4.096
H(35)	-7.855	-0.356	2.307
H(36)	-5.858	-1.46	3.103
H(37)	-4.12	-1.241	-0.752
H(38)	-4.657	-0.428	-2.903
H(39)	-5.64	0.551	-4.911
H(40)	-7.867	1.537	-4.842
H(41)	-9.15	1.565	-2.776
H(42)	-3.322	-2.903	1.019

H(43)	-3.818	-2.63	2.696

Supplementary Table 5. Input geometry of DBT-carbon-DBTSO fragments.

Atom	x	y	z
S(1)	0.396	1.798	2.283
C(2)	0.973	1.458	0.976
C(3)	0.339	0.442	0.372
C(4)	-0.658	0.029	1.162
C(5)	-0.664	0.781	2.272
C(6)	2.019	2.052	0.382
C(7)	2.416	1.61	-0.823
C(8)	1.778	0.592	-1.425
C(9)	0.73	-0.003	-0.831
C(10)	-1.557	-0.948	0.983
C(11)	-2.482	-1.18	1.934
C(12)	-2.481	-0.417	3.044
C(13)	-1.581	0.563	3.226
S(14)	-8.662	0.227	-0.085
C(15)	-7.147	-0.529	0.524
C(16)	-6.139	-0.669	-0.351
C(17)	-6.456	-0.197	-1.565
C(18)	-7.703	0.297	-1.606
C(19)	-7.007	-0.94	1.792
C(20)	-5.849	-1.496	2.181
C(21)	-4.829	-1.649	1.317
C(22)	-4.986	-1.228	0.048
C(23)	-5.692	-0.171	-2.67
C(24)	-6.185	0.348	-3.806
C(25)	-7.433	0.841	-3.843
C(26)	-8.198	0.817	-2.74
C(27)	-3.52	-2.267	1.758
O(28)	-9.724	-0.764	-0.191
O(29)	-8.836	1.559	0.478
H(30)	2.558	2.89	0.857
H(31)	3.275	2.088	-1.325
H(32)	2.117	0.241	-2.414
H(33)	0.213	-0.837	-1.332
H(34)	-1.546	-1.557	0.066
H(35)	-3.24	-0.595	3.824
H(36)	-1.613	1.168	4.149
H(37)	-7.831	-0.824	2.514
H(38)	-5.739	-1.827	3.227
H(39)	-4.146	-1.35	-0.653
H(40)	-4.664	-0.567	-2.672
H(41)	-5.561	0.371	-4.716
H(42)	-7.831	1.266	-4.779

H(43)	-9.223	1.222	-2.773
H(44)	-3.193	-3.024	1.008
H(45)	-3.636	-2.832	2.711

Supplementary Table 6. DFT-optimized geometry of DBT-carbon-DBT fragments, computed at the level of B3LYP/6-31G (d, p).

Atom	x	y	z
C(1)	5.026	1.949	0.852
C(2)	5.447	1.432	-0.315
C(3)	4.775	0.427	-0.903
C(4)	3.669	-0.079	-0.333
C(5)	3.253	0.441	0.83
C(6)	3.921	1.443	1.421
C(7)	2.2	0.122	1.591
C(8)	2.192	0.921	2.667
S(9)	3.31	1.874	2.685
C(10)	1.252	-0.808	1.41
C(11)	0.275	-0.943	2.326
C(12)	0.274	-0.134	3.403
C(13)	1.224	0.798	3.586
C(14)	-0.818	-1.974	2.148
C(15)	-2.072	-1.298	1.637
C(16)	-2.152	-0.912	0.35
C(17)	-3.263	-0.305	-0.089
C(18)	-4.297	-0.075	0.732
C(19)	-4.23	-0.452	2.017
C(20)	-3.115	-1.06	2.455
C(21)	-3.569	0.165	-1.303
C(22)	-4.801	0.695	-1.261
S(23)	-5.42	0.6	0.068
C(24)	-2.853	0.164	-2.436
C(25)	-3.396	0.708	-3.538
C(26)	-4.63	1.24	-3.5
C(27)	-5.343	1.238	-2.361
H(28)	5.594	2.774	1.316
H(29)	6.354	1.836	-0.796
H(30)	5.135	0.015	-1.86
H(31)	3.125	-0.902	-0.823
H(32)	1.266	-1.455	0.52
H(33)	-0.527	-0.233	4.155
H(34)	1.188	1.443	4.481
H(35)	-0.51	-2.774	1.436
H(36)	-1.001	-2.498	3.114
H(37)	-1.306	-1.095	-0.331
H(38)	-5.064	-0.274	2.718
H(39)	-3.059	-1.364	3.514
H(40)	-1.841	-0.269	-2.477

H(41)	-2.824	0.718	-4.481
H(42)	-5.061	1.683	-4.414
H(43)	-6.354	1.68	-2.353

Supplementary Table 7. DFT-optimized geometry of DBT-carbon-DBTSO fragments, computed at the level of B3LYP/6-31G (d, p).

Atom	x	y	z
C(1)	4.191	1.523	1.287
C(2)	3.562	0.499	0.692
C(3)	2.56	0.096	1.48
C(4)	2.545	0.862	2.58
S(5)	3.603	1.88	2.585
C(6)	5.242	2.11	0.694
C(7)	5.648	1.653	-0.502
C(8)	5.015	0.626	-1.096
C(9)	3.963	0.038	-0.502
C(10)	1.663	-0.885	1.307
C(11)	0.731	-1.105	2.253
C(12)	0.723	-0.328	3.353
C(13)	1.621	0.655	3.529
C(14)	-0.304	-2.196	2.084
C(15)	-1.615	-1.583	1.643
C(16)	-1.77	-1.155	0.376
C(17)	-2.926	-0.601	-0.023
C(18)	-3.938	-0.474	0.849
C(19)	-3.8	-0.894	2.114
C(20)	-2.64	-1.444	2.504
C(21)	-3.241	-0.121	-1.235
C(22)	-4.492	0.364	-1.278
S(23)	-5.456	0.275	0.239
C(24)	-2.473	-0.083	-2.336
C(25)	-2.964	0.442	-3.471
C(26)	-4.216	0.926	-3.509
C(27)	-4.985	0.888	-2.41
O(28)	-6.51	-0.724	0.123
O(29)	-5.641	1.601	0.812
H(30)	5.776	2.955	1.162
H(31)	6.51	2.124	-1.003
H(32)	5.361	0.263	-2.078
H(33)	3.451	-0.802	-0.996
H(34)	1.682	-1.505	0.397
H(35)	-0.041	-0.497	4.131
H(36)	1.581	1.271	4.444
H(37)	0.023	-2.955	1.336
H(38)	-0.416	-2.757	3.04
H(39)	-0.927	-1.266	-0.322
H(40)	-4.629	-0.788	2.834

H(41)	-2.533	-1.781	3.548
H(42)	-1.442	-0.471	-2.337
H(43)	-2.337	0.476	-4.377
H(44)	-4.612	1.356	-4.444
H(45)	-6.013	1.287	-2.444

Supplementary References

1. Ashby, J., Cook, C.C. Recent advances in the chemistry of dibenzothiophenes. In: *Advances in Heterocyclic Chemistry* (eds Katritzky AR, Boulton AJ). Academic Press (1974).
2. Che, Y., Ma, W., Ji, H., Zhao, J., Zang, L. Visible photooxidation of dibenzothiophenes sensitized by 2-(4-methoxyphenyl)-4, 6-diphenylpyrylium: An electron transfer mechanism without involvement of superoxide. *J. Phys. Chem. B* **110**, 2942-2948 (2006).
3. Chen, D., *et al.* Covalent organic frameworks containing dual O_2 reduction centers for overall photosynthetic hydrogen peroxide production. *Angew. Chem. Int. Ed.* **62**, e202217479 (2022).
4. Cheng, H., Lv, H., Cheng, J., Wang, L., Wu, X., Xu, H. Rational design of covalent heptazine frameworks with spatially separated redox centers for high-efficiency photocatalytic hydrogen peroxide production. *Adv. Mater.* **34**, 2107480 (2022).
5. Chang, J.-N., *et al.* Oxidation-reduction molecular junction covalent organic frameworks for full reaction photosynthesis of H_2O_2. *Angew. Chem. Int. Ed.* **62**, e202218868 (2023).
6. Chai, S., Chen, X., Zhang, X., Fang, Y., Sprick, R.S., Chen, X. Rational design of covalent organic frameworks for efficient photocatalytic hydrogen peroxide production. *Environ. Sci.: Nano* **9**, 2464-2469 (2022).
7. Shiraishi, Y., *et al.* Resorcinol–formaldehyde resins as metal-free semiconductor photocatalysts for solar-to-hydrogen peroxide energy conversion. *Nat. Mater.* **18**, 985-993 (2019).
8. Liu, Y., *et al.* Substoichiometric covalent organic frameworks with uncondensed aldehyde for highly efficient hydrogen peroxide photosynthesis in pure water. *Appl. Catal. B* **331**, 122691 (2023).
9. Chen, L., *et al.* Acetylene and diacetylene functionalized covalent triazine frameworks as metal-free photocatalysts for hydrogen peroxide production: a new two-electron water oxidation pathway. *Adv. Mater.* **32**, 1904433 (2020).
10. Chen, D., *et al.* Covalent organic frameworks containing dual O_2 reduction centers for overall photosynthetic hydrogen peroxide production. *Angew. Chem. Int. Ed.* **62**, e202217479 (2023).
11. Yuan, L., Zhang, C., Wang, J., Liu, C., Yu, C. Mesoporous resin nanobowls with optimized donor-acceptor conjugation for highly efficient photocatalytic hydrogen peroxide production. *Nano Res.* **14**, 3267-3273 (2021).
12. Zhang, K., Li, H., Shi, H., Hong, W. Polyimide with enhanced π stacking for efficient visible-light-driven photocatalysis. *Catal. Sci. Technol.* **11**, 4889-4897 (2021).
13. Zhou, Z., *et al.* A thioether-decorated triazine-based covalent organic framework towards overall H_2O_2 photosynthesis without sacrificial agents. *Appl. Catal. B* **334**, 122862 (2023).
