# Supplementary material for: In-situ formatting donor-acceptor polymer with giant dipole moment and ultrafast exciton separation
Source: Nat Commun. 2024 Feb 13;15:1313. doi: 10.1038/s41467-024-45604-5 (PMC10864376; doi:10.1038/s41467-024-45604-5)
Supplement: Supplementary file 3 — Description of Additional Supplementary Files [file 41467_2024_45604_MOESM3_ESM.pdf]

Description of Additional Supplementary Files

File Name: Supplementary Data 1

Description: Xyz coordinates of inputted DBT-carbon-DBT fragments.

File Name: Supplementary Data 2

Description: Xyz coordinates of inputted DBT-carbon-DBTSO fragments.

File Name: Supplementary Data 3

Description: Xyz coordinates of DFT-optimized DBT-carbon-DBT fragments.

File Name: Supplementary Data 4

Description: Xyz coordinates of DFT-optimized DBT-carbon-DBTSO fragments.
